# Comparison of Tolerability and Impact on Metabolic Profiles of Antiretroviral Regimens Containing Darunavir/Ritonavir or Darunavir/Cobicistat in Romanian HIV Infected Patients

**DOI:** 10.3390/biomedicines9080987

**Published:** 2021-08-09

**Authors:** Ruxandra-Cristina Marin, Delia Mirela Tiț, Oana Săndulescu, Adrian Streinu-Cercel, Simona Gabriela Bungău

**Affiliations:** 1 Department of Pharmacy, Faculty of Medicine and Pharmacy, University of Oradea, 410028 Oradea, Romania; rcfhm@yahoo.com (R.-C.M.); mirela_tit@yahoo.com (D.M.T.); 2 Doctoral School of Biological and Biomedical Sciences, University of Oradea, 410073 Oradea, Romania; 3 Department of Infectious Disease, “Carol Davila” University of Medicine and Pharmacy, 050474 Bucharest, Romania; oanasandulescu1@gmail.com (O.S.); astreinucercel@yahoo.com (A.S.-C.); 4 “Prof. Dr. Matei Balș” National Institute of Infectious Diseases, 021105 Bucharest, Romania

**Keywords:** darunavir, ritonavir, cobicistat, antiretroviral therapy, tolerability, safety, retrospective study

## Abstract

The management of the side effects caused by the antiretroviral therapy is one of the main problems facing clinicians. The patient’s tolerability and safety influence the success of the therapy. This retrospective study assesses the tolerability and impact on metabolic profiles of antiretroviral regimens containing darunavir/ritonavir (DRV/r) versus those containing darunavir/cobicistat (DRV/c), in routine clinical practice. The database of Prof. Dr Matei Bals of the National Institute of Infectious Diseases (INBI MB) was studied for the period 2017–2020, allowing the inclusion in the study of 462 HIV-infected patients who received the current regimen at least three months before evaluation. The following parameters were collected and analyzed: significant medical history, associated diseases, serum levels for profile evaluation: carbohydrate, lipidic, serum level of liver and pancreatic enzymes, serum markers of cardiac function, coagulation, and renal function. DRV/c (800 mg/150 mg, once daily) administrated in combination with other antiretroviral (ARV) in HIV-1 infected subjects proved to be better tolerated and with a lower impact on metabolic profile than DRV/r (600 mg/100 mg, twice daily). Patients in DRV/r group are significantly more at risk of developing, over time, side effects and metabolic impairments than those in DRV/c group, in all body functions studied, with statistically significant differences (*p* < 0.05) between the two groups. Laboratory data were correlated with patient’s demographic and clinical characteristics and statistically significant outcomes have been found, proving that a personalized regimen is needed to minimize the ART side effects and to maximize the success of therapy. The results of the study showed that DRV/c, associated with other antiretroviral drugs in the regimens of Romanian HIV infected subjects, have a more favorable metabolic profile than those containing DRV/r.

## 1. Introduction

Antiretroviral therapy (ART) has led to a significant reduction in mortality and morbidity among people infected with human immunodeficiency virus (HIV). In Romania, recent data show that 77% of people living with HIV (PLWH) receive antiretroviral therapy and 70% of them are in the viral suppression stage [1]. As therapeutic approach, since 2001, HIV^+^ patients in Romania have received free medication through national programs from the first stage of diagnosis, regardless of the level of CD4 T lymphocytes [2]. This has increased life expectancy for PLWH, but it brings with it another challenge–the PLWH are more likely to experience aging-related problems, like the healthy people, but also with the side effects caused by ART. Studies demonstrated that PLWH suffer for premature aging of the immune system, multi-morbidity being an often-used expression [3,4]. The risk factors for comorbidity are associated with both the infection per se or other viral, bacterial, fungal, parasitic co-infections, and with the toxicity of antiretroviral (ARV) drugs [5]. The traditional risk factors are added: aging, dyslipidemia, diabetes, lipids profile changes and lipodystrophy, obesity, smoking, or drug use are added [6]. Thus, the management of side effects caused by ART is one of the main problems facing clinicians. Treatment of HIV infection evolved from solving the disease-specific complications to finding the balance between maintaining long-term viral suppression and the risks of drug toxicity [7]. The patient’s tolerability and safety influence the success of the therapy, with a direct effect on the patient’s adherence. More than half of patients end up changing the treatment regimen because of the side effects [8,9,10].

According to the latest data, 65% of HIV^+^ patients in Romania are young adults aged between 25 and 40. A third of them make up the 1990s pediatric cohort, now 30–35 years old and another third of patients are over 40. Therefore, the majority of PLWH in Romania are therapeutically aged patients for whom the greatest challenge is to improve the quality of life and to reduce, as far as possible, the side effects of ARV [11].

Comparative studies between different regimens, which assess the tolerability and safety, without affecting effectiveness, are necessary to increase the success of ART. Protease inhibitors are part of the backbone of any regimen. All classes of anti-retroviral drugs (nucleoside reverse transcriptase inhibitors (NRTI), non-nucleoside reverse transcriptase inhibitors (NNRTI), protease inhibitors (PI), entry inhibitors (EI), integrase inhibitors (II), fusion inhibitors (FI), boosting drugs) are available for prescription by the Romanian clinicians. These are formulated either as single molecules or as fixed double, triple, or quadruple doses. The Standard of Care (SoC) for experienced patients is considered to be a triple regimen, three molecules from at least two different therapeutic classes [12]. For treatment-naive patients, the regimen should be carefully chosen to prevent virological and/or immunological failure.

The side effects produced by ARVs may affect any organ or body type of metabolisms. Most of them are well tolerated, but patients may experience minor side effects, from time to time. Although these symptoms pass after the first weeks of therapy, side effects influence patient’s quality of life. The most cited symptoms are gastro-intestinal–nausea, diarrhea, bloating; dermatological–eczema, sleep disorders, headache, dizziness, and hypersensitivity reactions. However, there is also an important category of serious side effects reported to varying degrees: lactic acidosis, impaired liver function and/or lipodystrophy (redistribution of adipose tissue), for NRTI; urticaria, Steven-Johnson syndrome, toxic epidermal necrolysis after NRTI administration; hepatotoxicity, dyslipidemia, hyperglycemia and resistance to insulin, hypercholesterolemia, hypertriglyceridemia, lipodystrophy, for PI [13].

Darunavir (DRV), as a PI, was authorized for marketing in 2006 in the United States and in 2007 in Europe. DRV is a treatment option for children over 3 years of age, adolescents and treatment-naive adults, HIV-1 infected, but also for treatment-experienced patients [14]. Many studies have demonstrated its effectiveness in viral suppression, in experienced HIV^+^ patients, DRV becoming an important part of ART. DRV is also recommended for therapeutically aged patients who no longer have treatment options, due to its increased potency in the presence of resistant mutations to other PIs [15]. It has a high affinity for the HIV-1 protease, forming a stable complex due to its flexible conformation and chemical interactions [16,17].

In treatment regimens, DRV (400 mg, 600 mg, 800 mg) is administered, once or twice daily, at the same time with low doses of ritonavir (RTV) 100 mg or cobicistat (COBI) 150 mg. Enhancement of PI with low doses of enzyme inhibitors has significant clinical benefits, improving the pharmacological properties of PIs, especially by acting on the pharmacokinetics, absorption, and metabolism. The ability of RTV or COBI to increase the minimum plasma concentrations (Cmin) of PI by inhibiting the P 450 cytochrome (CYP450) when co-administrated is probably the greatest clinical benefit of boosted therapy. Inadequate antiretroviral concentrations would lead to therapeutic failure and the emergence of resistant viral variants [18]. ARV combinations are more likely to be better tolerated precisely by maintaining the plasma concentration of the drug in the therapeutic area. The effect is kept constant for a longer period without fluctuation between the minimum effective concentration (Cmin) and the maximum concentration (Cmax), above which the risk of toxicity would increase.

RTV and COBI similarly inhibit CYP3A4 and can be used with the same therapeutic efficacy, but COBI is preferred due to its higher specificity and the absence of inductive properties. The two boosters interact differently with other drugs metabolized by the enzymes on which they act [19,20]. RTV, once used as PI, has a high affinity for several CYP450 isoenzymes, strongly inhibits CYP3A4, as well as CYP2D6, CYP2C19, CYP2C8 and CYP2C9 [21]. RTV is an enzymatic inducer for CYP1A2, CYP2C19, CYP2C8, CYP2C9, CYP2B6, UGT1A4 [22]. However, it has tolerability problems for a large number of patients with various side effects either because of its own toxicity or due to drug–drug interactions when co-administrated [23].

COBI is a newer molecule that has been developed and used only for its pharmacokinetic boosting effect. Compared to RTV, COBI has no antiviral activity or inductive properties, being much more selective as an enzyme inhibitor and with fewer drug–drug interactions [20]. Another advantage for COBI is that it has a better solubility, affording coformulation with other ARV as simplified fixed dose combinations. DRV with COBI (DRV/c) reaches plasma concentrations bioequivalent to those obtained in co-administration with RTV (DRV/r) [24,25]. Studies have shown that plasma levels of DRV are 30% lower in co-administration with COBI than with RTV, which recommends COBI as enhancer, reducing the risk of side effects [24].

This real-life study aimed to compare the tolerability and the impact on metabolic profile of the schemes in which a PI like DRV, enhanced with either RTV or COBI is administered, answering a few questions:

- Is COBI a safer and better tolerated enhancer, with fewer side effects than RTV, in long-term administration?
- Which one of the two enhancers, COBI or RTV, has a stronger impact on the metabolic profile?
- When one enhancer or another is administered, the differences in the change of metabolic markers are sufficiently relevant and/or statistically significant to tip the balance in favor of using one of them (COBI or RTV)?

We also followed the role played by the patient’s history/particularities (e.g., age, comorbidities, duration of infection, duration of therapy, pill’s burden, etc.) and personal response to therapy in choosing the regimen, implicitly the enhancer. The numerous results obtained in this research allow the clinicians to perform an optimized management of the personalized PLWH therapy.

## 2. Materials and Methods

### 2.1. Design Study

This retrospective, observational, open, non-interventional study included 462 HIV–1 infected patients, consecutively enrolled, from the database of the ”Prof. Dr. Matei Bals” National Institute of Infectious Diseases (INBI MB), Romania. The study was performed from 2017 to 2020, the patients being divided into two groups and evaluated in routine clinical practice. One group received DRV boosted with RTV (600 mg/100 mg, twice a day), the second DRV boosted with COBI (800 mg/150 mg, once a day), co-administrated with other ARVs. HIV-infected male and female patients, over 18 years old, receiving in current practice boosted DRV with one of the two enzyme inhibitors (RTV or COBI), with clear indication of the DRV have been criteria for inclusion in the study. The current antiretroviral regimen has been administrated for at least three months before evaluation. 384 subjects (83.11%) were part of the first group (DRV/r) and 78 patients (16.88%) represented group 2 (DRV/c). The large difference in the number of patients between the two groups (384 vs. 78) is explained by the fact that RTV came into use in 1996 (so long ago), while COBI is a much newer enhancer (2017), used in antiretroviral therapy combined with DRV in a single tablet fixed-dose (called Rezolsta). In combination with the two ARVs, other antiretroviral drugs were part of the regimen: NRTI, NRTI, PI, II, or EI. Regimens without boosted DRV, patient’s lack of knowledge, age under 18, lack of patient’s medical evaluations, obvious clinical signs of organ decompensation, pregnancy, and lactation were exclusion criteria.

Clinical parameters investigated in the study were evaluated in routine clinical practice at INBI MB during treatment. They were collected and analyzed the following: vital signs, clinical, significant medical history, associated diseases, serum levels for profile assessment: carbohydrate (glycemia, hemoglobin glycosylate), lipid (total cholesterol), high density lipoprotein (HDL-cholesterol), low density lipoprotein (LDL-cholesterol), triglyceride (TG), serum level of liver enzymes (alanine aminotransferase–ALT/GPT, aspartate-aminotransferase–AST/GOT, gamma-glutamyl transferase–GGT, alkaline phosphatase–ALP, total bilirubin, direct bilirubin), and pancreatic enzymes (amylase and lipase) range, serum markers for cardiac function (creatine kinase (CK), CK–MB), coagulation (prothrombin time/international normalized ratio (PT/INR) and fibrinogen) and for the evaluation of renal function (uric acid, creatinine, serum urea, creatinine clearance). The blood samples were collected in the morning after a 12–14-h meal break.

The side effects considered as major reactions affecting body functions or types of metabolism were hyperglycemia, hypercholesterolemia, hypertriglyceridemia, increased liver and pancreatic enzymes, changes in cardiac and coagulation markers, renal impairment. To assess the profile of the patient at risk of undergoing metabolic and body organ damages, the values of the biochemical parameters have been statistically correlated with the demographic and disease-specific ones, as they were collected from patients’ medical records.

For using the INBI MB database, the agreement of the INBI MB’s management (9/5861, dated 4 May 2021) and the Bioethics Commission (C05865/4 May 2021) waiver of informed consent was obtained. The study was conducted in accordance with the ethical principles set out in the Helsinki Declaration and with the Good Clinical Practice guidelines [26,27].

### 2.2. Biochemical Determinations

Serum blood glucose level was determined à jeun (basic) for all subjects, using the spectrophotometric method (colorimetric enzymatic), using THE VITREOUS^®^ 5.1 FS Chemistry System and VITROS^®^ 4600 Chemistry System, manufacturer Orto Clinical Diagnostics Inc., New York, NY, USA. Venous blood was collected, and serum was analyzed. The results have been interpreted according to American Diabet Association (ADA) criteria. For glycosylated hemoglobin, turbidimetry was used as a working method, using the same analyzer. The entire cohort was evaluated, the laboratory determinations being made from blood on ethylene diamino tetra acetic acid (EDTA). Glycosylated hemoglobin is used as a marker of long-term blood glucose control.

The lipid profile of all patients was evaluated by determining serum levels of total cholesterol (TC), LDL-cholesterol, HDL-cholesterol, triglycerides (TG), very-low-density lipoprotein (VLDL), and total lipids (TL). Except for total lipids (TL) and VLDL, all other lipid parameters were measured by spectrophotometry, using VITROS^®^ 5.1 FS Chemistry System and VITROS^®^ 4600 chemistry System, manufacturer Orto Clinical Diagnostics Inc., New York, NY, USA. For calculating the serum level of TL, the following formula was used

LT = 2.25 × total cholesterol value + TG value + 90
(1)

and for calculating VLDL-cholesterol level it was used TG value/5. Cardiac markers, creatin kinase (CK) and creatin kinase isoenzyme MB (CK MB) have been measured from blood serum, spectrophotometrically, on VITROS^®^ 5.1 FS chemical System and VITROS^®^ 4600 Chemistry System, manufacturer Orto Clinical Diagnostics Inc., New York, NY, USA.

The coagulation markers evaluated in the study (PT/INR) and fibrinogen) have been determined from plasma, pre-incubated, using the automated analyzer Symex CS-2000i manufactured by Siemens Healthcare GmbH, Erlangen, Germany. This is an in vitro diagnostic tool that uses the coagulometric, chromogenic, and immunological detection method. Biochemical parameters for evaluation of liver function ALT/GPT, AST/GOT, GGT, ALP, total bilirubin, and direct bilirubin and those characteristics for the pancreatic function (amylase and lipase) were evaluated spectrophotometrically from a serum sample.

Spectrophotometry is the method by which the serum levels of the parameters of the renal function have also been determined: urea, uric acid, and creatinine. The samples were collected from serum and performed on THE VITROS^®^ 5.1 FS chemical System and VITROS^®^ 4600 Chemistry System, manufacturer Orto Clinical Diagnostics Inc., New York NY, USA. For the measurement of creatinine clearance, the Modification of Diet in Renal Disease (MDRD) study equation was used, thus estimating the glomerular filtration rate (GFR). This equation includes inputs based on: sex, age, serum creatinine and (optional) race [28]. The MDRD equation cannot be used for acute renal failure.

Interpretation of the results was performed according to Kidney Disease Improvement Global Outcomes (KDIGO) guide, for chronic kidney disease (CKD) [29].

### 2.3. Statistical Analysis

Due to the exploratory nature of the study, non-interventional, a descriptive statistical analysis has been carried out, using the SPSS Statistics 20.0.0 program (SPSS Inc., Chicago, IL, USA). For all parameters used in the study, descriptive analysis has been performed by calculating mean, standard deviation (SD), minimum value and maximum value for variables with normal distribution, and for non-parametric distribution the Mann–Whitney test was applied in SPSS Statistics. For qualitative parameters, the frequencies, the value number (COUNT) and the percentages of each category were presented. The descriptive analysis was carried out for the entire cohort, but also separately for each group. One or more of the following statistical methods have been used: the Pearson correlation coefficient for the quantitative comparison study between two variables (clinical parameters, two groups, or demographic data versus clinical evaluations); Student’s *t*-test, expressed by the coefficient t, for two independent samples (groups) or pairs, independent in our case; analysis of variance (ANOVA) F, to check the influence of qualitative factors, explained by the coefficient F of the ANOVA statistical test, for three or more independent groups or pairs, in our case independent. The statistical significance considered was *p* < 0.05. The Pearson’s correlation coefficient was used to associate population variables and demographic or disease-specific parameters. It was considered as a significant statistical result *p* value < 0.05. The sign “−“ indicates inversely proportiona correlation between the two parameters concerned, and the “+”values indicate a directly proportional link.

## 3. Results

### 3.1. Demographic and Clinical Characteristics

**Figure 1:**
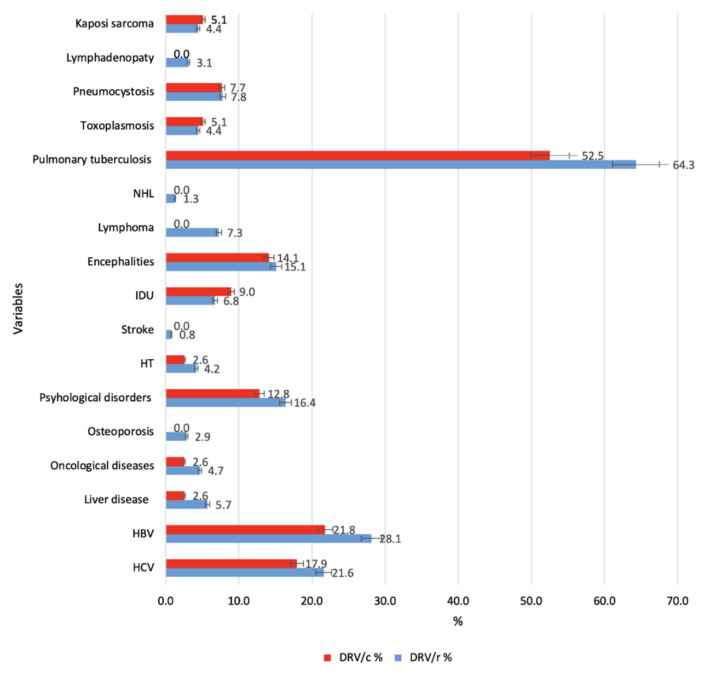
HIV-1 related comorbidities compared to the two groups (defined clustered bar: summaries of group of cases (% of cases). Legend: NHL—non-Hodgkin lymphoma; IDU—injection drug use; HT—hypertension; HBV—hepatitis B virus; HCV—hepatitis C virus.

In terms of demographic characteristics and according to the classification of HIV disease and infection, made by the United States Agency, the Centers for Disease Control and Prevention (CDC), there are no statistically significant differences between the two groups (Table 1). A significantly higher number (F = 4.662. *p* = 0.031) of comorbidities was noticed in patients in group 1 (DRV/r), then in group 2 (DRV/c), as it is depicted in Figure 1.

**Figure 2:**
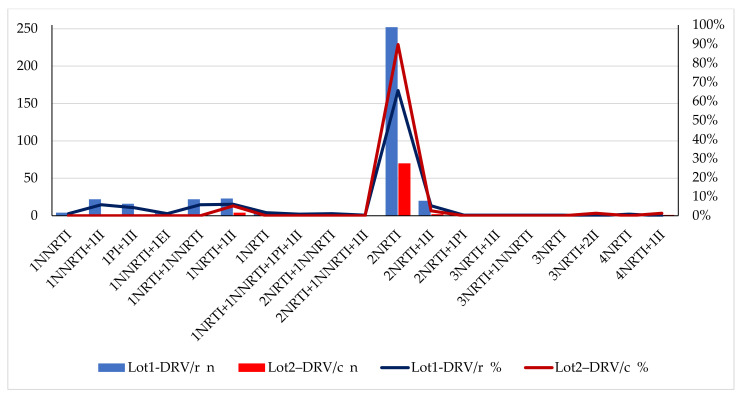
Therapeutic classes associated with DRV/r or DRV/c compared to the two groups (Excel, Microsoft). Legend: NRTI—nucleoside reverse transcriptase inhibitor; NNRTI—non-nucleoside reverse transcriptase inhibitor; PI—protease inhibitor; II—integrase inhibitor; EI—entry inhibitor; n—number of patients in group.

The description of the cohort, according to associated ARV and as number and type of therapeutic classes, for each group, is shown in Figure 2.

Most patients in both groups (60.15% from DRV/r and 79.47% from DRV/c) received the same three regimens, the difference being represented only by the enhancer (Table 2).

### 3.2. Biochemical Assessments of Tolerability and Safety

#### 3.2.1. Carbohydrate Metabolism

**Figure 3:**
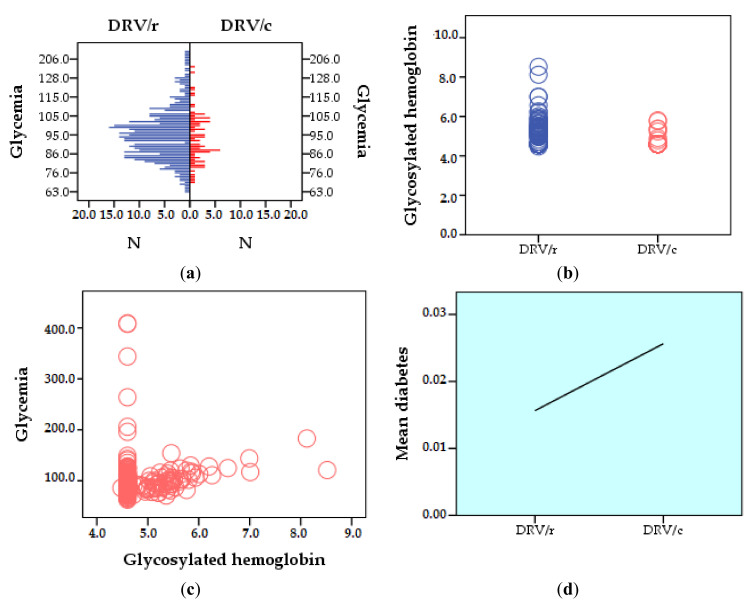

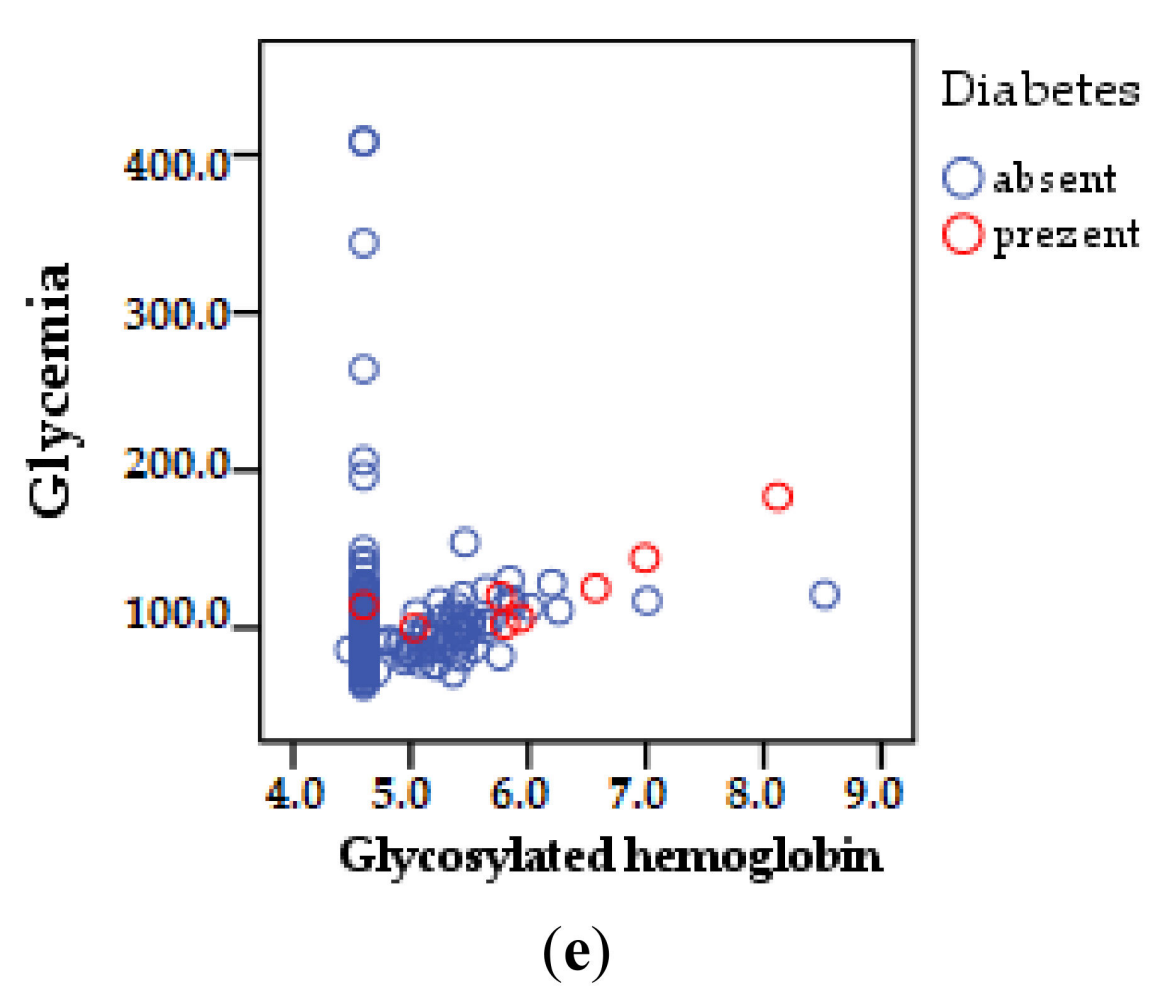
Comparative carbohydrate profile between groups: (**a**) glycemia (mg/dL); (**b**) glycosylated hemoglobin (%); (**c**) Pearson correlation between blood glucose and glycosylated hemoglobin; (**d**) Pearson correlation regarding the prevalence of diabetes; (**e**) Pearson correlation pre-existing diabetes, blood sugar, and glycosylated hemoglobin (comparative histogram and dot cloud, SPSS).

In the group exposed to DRV/r, approximately 8% of patients have above normal blood glucose values (>115 mg/dL) and 12.72% have normal/high values (105–115 mg/dL). For patients in group 2, DRV/c, the blood glucose values are within normal limits (75–115 mg/dL) for 97.44% of the subjects, the remaining of 2.56% having high values (Figure 3a). According to the values of glycosylated hemoglobin, in the group exposed to DRV/r, 5 patients have diabetes (>6.5%) and 15 patients have prediabetes (5.6–6.4%) condition. Most patients in group 2 have values that are concentrated in references ranges (4.8% and 5.5%). Only two patients, 2.6%, of those exposed to DRV/c were found in the prediabetes condition (Figure 3b). The Pearson correlation for the two parameters, glycemia and glycosylated hemoglobin has statistical significance (r = 0.144, *p* = 0.002) (Figure 3c). Statistical analysis showed a mean value of the prevalence of pre-existing diabetes of 0.0156 (SD ± 0.012) for the first group and a higher mean value of 0.0256 (SD ±0.1590) for the second group, with statistically significant difference (*p* < 0.05) (Figure 3d). Correlating with the Pearson coefficient, the three parameters (pre-existing diabetes, serum blood glucose levels, and glycosylated hemoglobin) results indicates a strong positive correlation, as follows: pre-existing diabetes and blood glucose level (r = 0.120, *p* = 0.010); pre-existing diabetes and glycosylated hemoglobin (r = 0.443, *p* = 0.001) (Figure 3e).

#### 3.2.2. Lipid Metabolism

The parameters that describe lipid metabolism have been assigned the value 1—if they fall within normal limits; 0—if they have values below the limit; and 2—if they have values above the limit.

A higher number of patients with HDL-cholesterol values below the reference range were found in group one (DRV/r) than in group 2 (DRV/c) (*p* < 0.01). For LDL-cholesterol (NV—60–130 mg/dL), the mean values for the DRV/r group are 1.58 (SD ±0.493). The mean LDL-cholesterol values are 1.50 (SD ±0.503), for the DRV/c group, half of the patients exceeding the normal values. More than half (56.2%) of the patients in the group exposed to DRV/r and 43.6% of those exposed to DRV/c, had TG values above the limit (NV- <150 mg/dL). Comparing statistically the outcomes obtained for the two groups, a mean value of 1.56 (SD ±0.49) is observed for DRV/r and 1.43 (SD ± 0.49) for DRV/c (*p* < 0.05). For total lipids (TL), (NV- 500–800 mg/100 mL blood), the mean value is 1.35 (SD ±0.47) for group 1, and 1.21 (SD ±0.41) for group 2. The difference between the groups is more pronounced for VLDL-cholesterol (NV 2–38 mg/dL). 21.6% patients with higher values in DRV/r group (mean value 1.35, SD ± 0.47) than 9% patients with laboratory outcomes above the reference range, in the DRV/c group (NV 2–38 mg/dL), mean value of this group being 1.21 SD ± 0.41 (*p* < 0.01) (Table 3)

The outcomes obtained when analyzing the parameters that describe the carbohydrate metabolism (glycemia and glycosylated hemoglobin) and the lipid metabolism (total cholesterol, HDL-cholesterol, LDL-cholesterol, TG, VLDL-cholesterol, and total lipids) were cumulated to determine the patient’s profile at risk of cardiovascular damage, while taking ARV. The results indicated that

- CT level is strongly positively correlated with age, *p* < 0.01;
- HDL-cholesterol strongly correlates negatively with the number of years of therapy. The same type of relationship, inversely proportional to the number of ARVs in a scheme;
- LDL-cholesterol correlates positively, *p* < 0.05, increase directly proportional to the number of comorbidities;
- total lipids correlate directly proportionally, strongly positive (*p* < 0.01) with age and positive (*p* < 0.05) with the number of years of therapy;
- the level of VLDL-cholesterol is directly proportional to age, number of years of therapy and number of schemes (strongly positive correlation *p* < 0.01) but also to the number of comorbidities (positive correlation *p* < 0.05);
- strong positive correlation *p* < 0.01 also exists between glycosylated hemoglobin and age, number of years of therapy, comorbidities, number of ARVs;
- blood glucose value was significantly correlated, strongly positive *p* < 0.01 only with the age of patients (Table 4).

Comparing the prevalence of lipodystrophy in the two groups, the results showed insignificant differences (35.4%-DRV/r vs. 33.3%-DRV/c). Pre-existing dyslipidemia (before the present regimen) occurs in 28.6% of patients in the DRV/r group, compared to 15.3% in the DRV/c group (Table 5).

**Figure 4:**
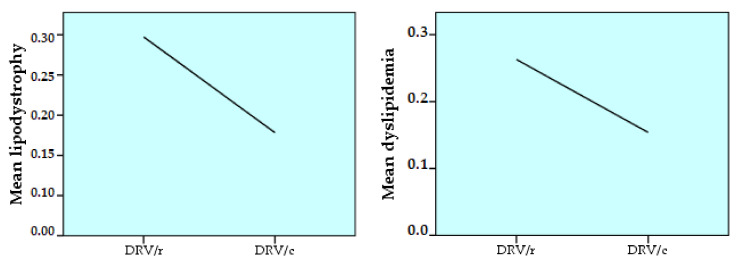
Pearson correlation between group 1 (DRV/r) and group 2 (DRV/c), regarding the prevalence of pre-existing lipodystrophy and dyslipidemia (SPSS, Line Area).

Comparing the number of pre-existing cases of lipodystrophy and dyslipidemia (before the present regimen) in the two groups and applying the Pearson coefficient, line charts are obtained from Figure 4.

The prevalence among the studied cohort of diabetes, lipodystrophy and dyslipidemia pre-existing the current treatment regimen was considered to determine the risk of the HIV^+^ patient to develop these conditions throughout life, when treated with ARV. The results showed a statistically significant difference only for lipodystrophy and dyslipidemia:

- lipodystrophy is strongly positively correlated (*p* < 0.01), directly proportionally, with age. It also correlates positively (*p* < 0.05) with the number of comorbidities;
- dyslipidemia is strongly positively correlated with age, ART duration and number of comorbidities (*p* < 0.01). A positive correlation (*p* < 0.05) occurs between dyslipidemia and the number of ARVs in the regimen or the number of past regimens (Table 6).

#### 3.2.3. Cardiac and Coagulation Markers

**Figure 5:**
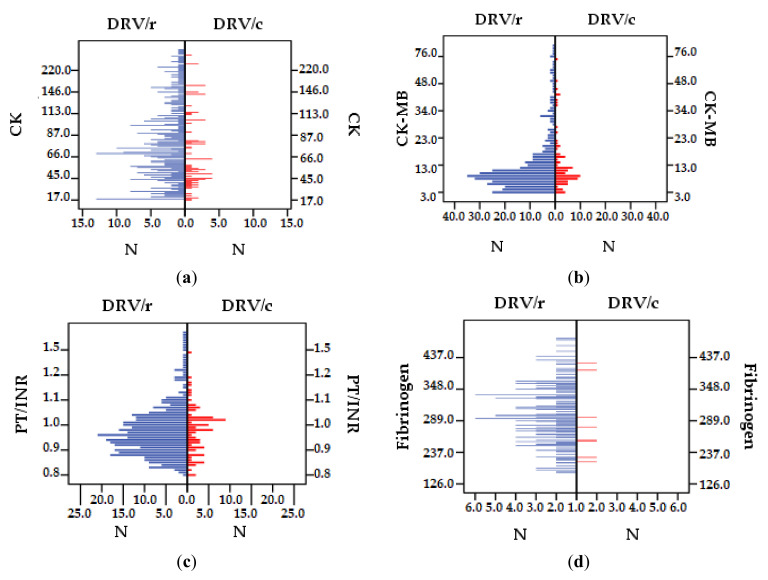
Difference between DRV/r and DRV/c as cardiac and coagulation markers: (**a**) CK (IU/L), (**b**) CK-MB (IU/L), (**c**) PT/INR, (**d**) fibrinogen (mg/mL).

Regarding CK, NV < 170 IU/L, the highest value exceeds 329 IU/L for several patients exposed to DRV/r (Figure 5a). The isoenzyme CK-MB (NV 2–10 IU/L) exceeds the reference range, in both groups, but the highest value was registered in DRV/r group (Figure 5b). PT/INR (NV 0.8–1.18) has higher values at DRV/r than DRV/c, but the mean value is within reference range (Table 7). However, in group 1 there are several patients (2.60%) with values above normal, with a maximum of 3.45, while in DRV/c group there is only one patient with values above the normal limit, 1.58 (Figure 5c). For fibrinogen (NV 200–400 mg/dL^2^) the most dangerous values are those <100 and >700. The lowest value (126 mg/dL^2^) as well as the highest (738 mg/dL^2^) are found in DRV/r group (Figure 5d). All patients exposed to DRV/c have serum fibrinogen values within normal limits (Table 7).

The Pearson correlation between the markers specific to cardiac function and coagulation with the characteristic parameters of the cohort, reached the statistically significant threshold only in the case of fibrinogen (fibrinogen correlates directly proportionally, strongly positively with age, *p* < 0.01) (Table 8).

#### 3.2.4. Liver Function

Majority (86.1%) of DRV/r patients have values >30 IU/L (NV 10–30 IU/L) for hepatic ALT, the mean value 36.62 IU/L also exceeding the normal limit. In the DRV/c group only 13.9% of the subjects exceed the reference range, but the mean value (29.02 IU/L) is found in the reference range. For AST, 85.5% of the patients in the DRV/r group show increased outcomes, the mean value being 34.94 IU/L. Only 14.5% of the patients exposed to DRV/c, have over the limit laboratory outcomes, the mean value being 29.27 IU/L.

In DRV/r group, GGT presents normal-high values for 88% of patients compared to the group DRV/c where only 12% patients have values near the upper limit of the reference range, but not exceeding it (NV < 60 IU/L). Regarding ALP, none of the patients in the cohort exceeds the reference range, (NV < 240 IU/L for women and <270 IU/L for men). The mean value is 82.69 IU/L in DRV/r group, higher than values in DRV/c group, 76.96 IU/L. The mean value of total bilirubin (NV 0.3–1.3 mg/dL) does not exceed the reference range in any of the two group. Still, the highest and lowest value was found in DRV/r group (24.60 mg/dL, respectively <0.2 mg/dL). For direct bilirubin (NV < 0.3 mg/dL), it was observed that the mean level is higher in DRV/r group, but overall laboratory outcomes remain within reference range.

Pancreatic amylase (NV 30–100 IU/L) has increased values in both groups. In the DRV/r group, 42 patients (10.93%), exceeded the normal range (over 100 IU/L). In DRV/c the mean value is lower than in DRV/r, with only two patients having values above normal (2.56%).

**Figure 6:**
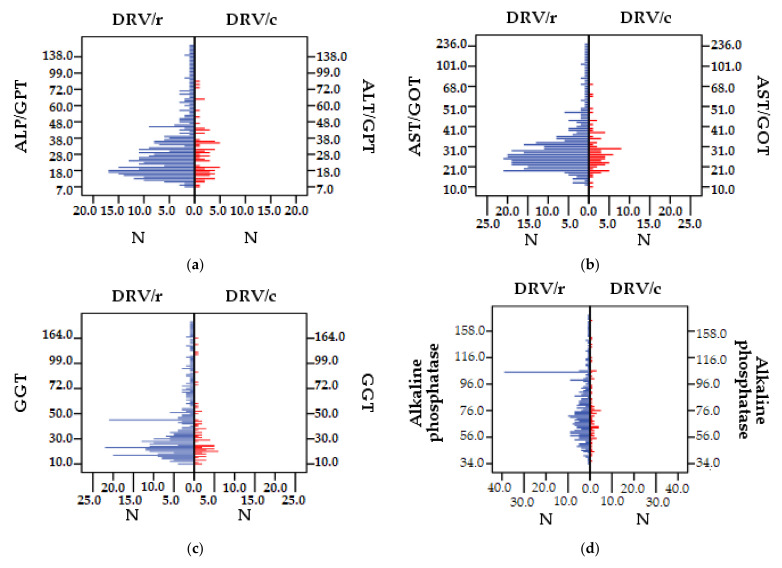

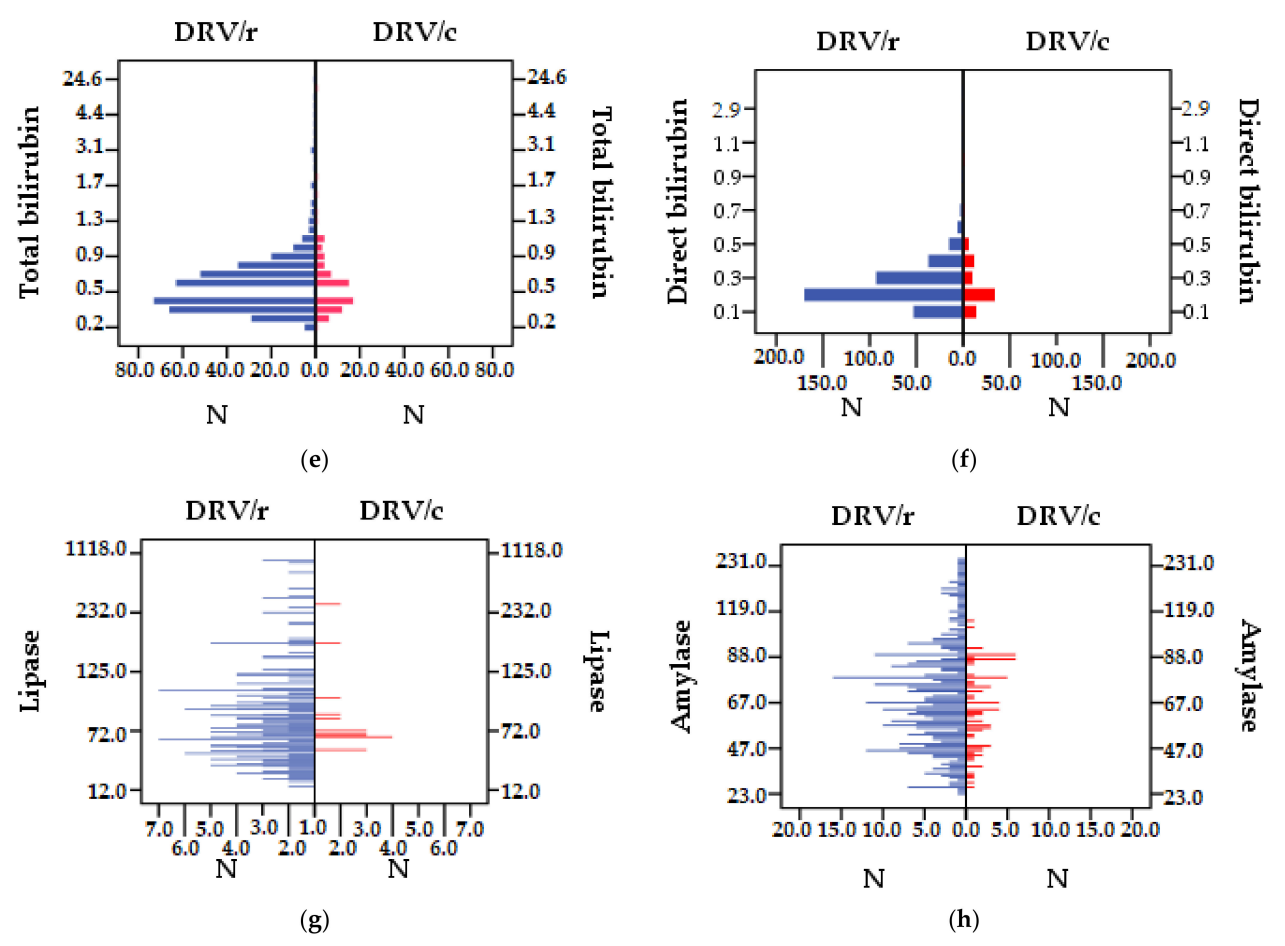
Difference between DRV/r and DRV/c groups as values of liver and pancreatic function parameters: (**a**) ALP/GPT (IU/L), (**b**) AST/GOT (IU/L), (**c**) GGT (IU/L), (**d**) IU/L), (**e**) total bilirubin (mg/dL), (**f**) direct bilirubin (mg/dL), (**g**) lipase (IU/L), (**h**) amylase (IU/L).

For seric lipase (NV 23–300 IU/L), in the DRV/r group, 8.59% of patients had values above normal limits compared to 5.12% in the group exposed to DRV/c. However, the mean values fall within the reference range. Outcomes <23 IU/L were found for only three patients in the entire cohort (two in the first group, and one in the second group). Descriptive statistics of liver and pancreatic function parameters are presented in Table 9 and the distribution of the values of the analyzed parameters, compared between group in Figure 6a–h.

Following the Pearson correlation between the values of liver and demographic parameters, the results are presented in Table 10. Co-infections with B and C hepatic viruses were added to the initial liver parameters, directly influencing the degree of liver function.

The following statistically significant correlations were observed:

- ALT/GPT correlates directly proportionally, strongly positively (*p* < 0.01) with the number of comorbidities and co-infection with VHC and positively (*p* < 0.05) with the number of treatment regimens and pills burden;
- AST/GOT is strongly positively correlated (*p* < 0.01) with VHC co-infection;
- GGT with HCV co-infection correlates directly proportionally, strongly positive, (*p* < 0.01);
- total bilirubin is positively correlated (*p* < 0.05) also with co-infections with VHC;
- direct bilirubin correlates strongly positively (*p* < 0.01) with VHC co-infection;
- pancreatic lipase is correlated directly proportionally, strongly positive (*p* <0.01) with the number of therapeutic schemes and positive (*p* <0.05) with age and duration of therapy.

#### 3.2.5. Renal Function

**Figure 7:**
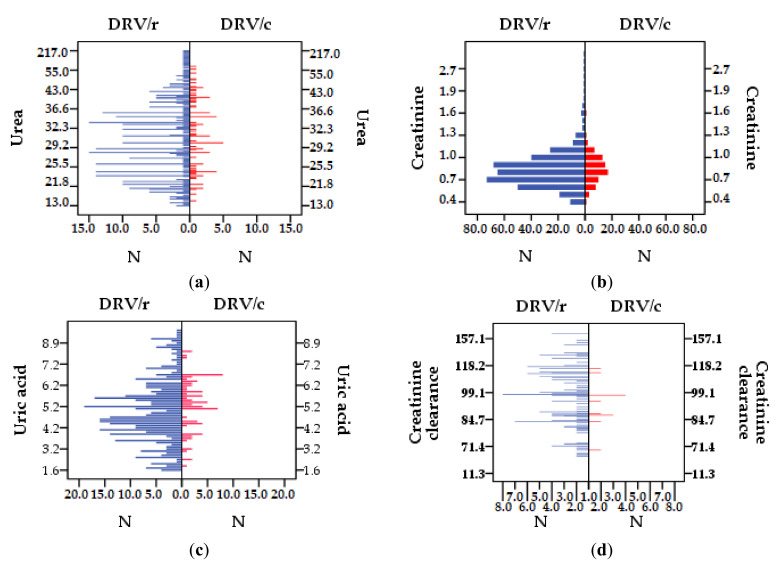
Distribution of parameter values for the entire cohort. Difference between batches, DRV/r and DRV/as renal function parameter values: (**a**) urea (mg/dL), (**b**) creatinine (mg/dL), (**c**) uric acid (mg/dL), (**d**) creatinine clearance (mL/min).

There were patients receiving DRV/r) who had higher serum urea (blood urea nitrogen) values (>45 mg/dL) than those in the DRV/c group, even if the mean values were <45 mg/dL. The explanation is that, although the mean values are very close (33.25 and 32.52) for the two groups, the standard deviation for the two groups is very different (SD = 18.61 in the group DRV/r and SD = 8.60 in the DRV/c group) (Table 11), which justifies the presence of higher urea values in the DRV/r group. The lowest value (13 mg/dL) but also the highest (2 patients > 200 mg/dL) are found in the DRV/r group. Only three patients exposed to DRV/c had values slightly above the normal limit, 60 mg/dL (Figure 7a). The mean value for serum creatinine is higher in the regimen containing DRV/r compared to DRV/c (NV < 1.1 mg/dL). In the DRV/r group there are several patients above the normal limit, the highest value being 12.6 mg/dL, while in the DRV/c group there is only one patient who exceeds the limit, reaching 1.6 mg/dL (Figure 7b). For uric acid (NV for women 2.5–7 mg/dL and 3–9 mg/dL for men) in the DRV/r group a higher fluctuation of the laboratory outcomes can be observed <1.6 mg/dL and >10.20 mg/dL (Figure 7c). Thus, the mean value for this group is lower than in the case of DRV/c, 5.01 mg/dL respectively 5.31 mg/dL. Within the DRV/c group, the distribution of uric acid values is mainly within the normal limits (tables).

The mean value for creatinine clearance is in the reference range (95–150 mL/min) in both groups, the lowest value belonging to DRV/c, 11.30 mL/min, and the highest one (226 mL/min) to the group DRV/r. Of the 26 patients with values above the normal limit, only 2 of them belong to DRV/c group (Figure 7d).

**Figure 8:**
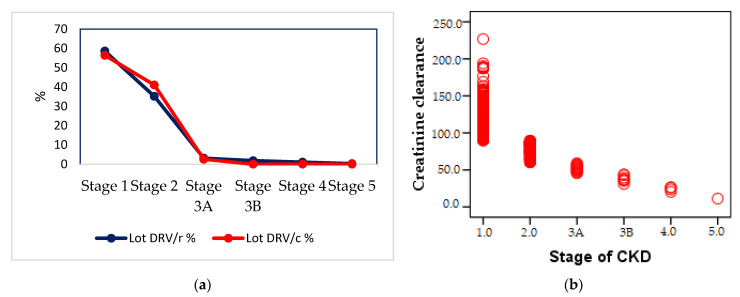
(**a**) Percentage distribution of renal impairment in the population of groups, (**b**) Pearson correlation between the value of renal clearance and the impairment of renal function.

Most patients in both groups are in stage I (58.59% DRV/r and 56.41 DRV/c) and stage II (35.15% DRV/r and 41.02% DRV/c) of renal impairment (normal and high GFR > 90 mL/min, respectively GFR 60–89 mL/min), without statistically significant (*p* < 0.05) differences between the two groups. In stage 3A, moderate impairment (GFR 45–59 mL/min), there is a small number of patients in both groups 12 (3.12%) in the DRV/r group, and 2 (2.56%) in the DRV/c group. For advanced stages of renal impairment, 3B (moderate impairment with GFR 30–44 mL/min), 4 (severe impairment with GFR = 15–29 mL/min) and 5 (final stage, GFR < 15 mL/min), there are patients only in the first group-3B−1.82%, 4–1.04% and 5–0.26% of patients (Figure 8a). The stage of renal impairment is inversely proportional to the creatinine clearance value (r = −0.769, *p* = 0.001) (Figure 8b).

The results of the statistical correlation between the parameters specific to renal function and those characteristics of the cohort showed that:

- urea correlates strongly positively, *p* < 0.01, with the age of patients;
- creatinine correlates positively, directly proportional, *p* < 0.05, with the age of patients;
- uric acid is strongly positively correlated, *p* < 0.01 with the age of patients and the number of comorbidities;
- creatinine clearance is directly proportional, strongly positively correlated, *p* < 0.01 with the duration of ART;
- the stage of renal impairment is strongly positively correlated, *p* < 0.01, with the duration of ART (Table 12).

## 4. Discussion

On January 1, 2017, UNAIDS launched its proposed targets for HIV infection management. These were: 90% of people diagnosed, 90% of those diagnosed to receive antiretroviral treatment and 90% of those taking ART to be under viral suppression. Later, another target was added [30]. In 2019, the annual report on HIV/AIDS Infection in Romania mentioned this fourth target, the concept of long-term health-90% of HIV^+^ patients who have undetectable viremia, under treatment with ARV, to have a quality of life similar to seronegative people [11]. This goal requires rigorous management of the side effects caused by the anti-retroviral therapy, taking into consideration the specific symptoms of aging.

Although the characteristics of the HIV^+^ cohort in Romania are unique in Europe due to the age at which patients were infected and the HIV-1 F-subtype [31], the implications of aging remain the same. Several observational studies carried out, on the Romanian cohort, have shown the impairment of various types of metabolism and human body systems caused by ART and the risk of comorbidities to which patients in the pediatric cohort of the 1990s and recently infected patients are exposed [31,32]. The toxicity of antiretroviral drugs remains the main reason for changing regimens [33]. For this reason, clinicians are constantly looking for simplified regimens which ensure, in addition to favorable virological and immunological responses, the highest possible degree of safety and tolerability. The present study aims to compare the tolerability and the impact on metabolic profiles of two antiretroviral regimes that contained, among other ARVs, DRV (PI) boosted with one of the two currently existing enzyme inhibitors, RTV or COBI. The study analyzed biochemical parameters specific to five basic human body types of metabolism: carbohydrate, lipid, cardiovascular, hepatic, and renal. Overall, the results showed that the therapeutic regimen containing COBI as pharmacokinetic enhancer is better tolerated and has a lower impact on the metabolic profile compared to that containing RTV.

Regarding carbohydrate metabolism, a higher incidence of high blood glucose levels was observed, >115 mg/DL in group 1 (DRV/r) compared to group 2 (DRV/c), forming a compact group, even though a smaller percentage of the patients in DRV/r group was diagnosed with pre-existing diabetes. The prevalence of pre-existing diabetes is higher at DRV/c, but the results of paraclinical analysis show a lower impairment (only 2.5% have blood glucose above normal level) in this group, compared to 8% at DRV/r. 2.6% have glycosylated hemoglobin in the prediabetes area (in DRV/c group) compared to 5.2% at DRV/r diabetes and pre-diabetes area. There is a proportional increase between the three parameters—the higher the prevalence of diabetes in the study population is, the higher the incidence of hyperglycemia and higher values of glycosylated hemoglobin are.

The Pearson correlation also indicates a strong positive relationship between blood sugar and glycosylated hemoglobin. The higher the blood sugar levels, the higher the level of glycosylated hemoglobin, in all patients in the studied cohort (DRV/r + DRV/c). Although, nowadays, the PLWH live longer than they did 30 years ago, they have several unique risk factors for metabolic disorder, including diabetes: ART, older generations of protease inhibitors or reverse transcriptase inhibitors, weight gain, lipodystrophy, and co-infection with VHC [34].

A study carried out on the Multicenter AIDS Cohort Study (MACS), in 2005, found that the incidence of diabetes is four times higher in HIV^+^ men and the prevalence of diabetes is 14% among men taking ART compared to 7% of men not taking ART despite they are infected, while only 5% of seronegative men suffer from diabetes [35].

Previous studies have also shown that in HIV^+^ patients receiving ART, hyperglycemia is caused by insulin resistance, as in type II diabetes. The mechanism is related to the impairment of glucose the transport by therapy, and/or the influence of intracellular glucose phosphorylation. Hyperglycemia occurs frequently in regimens containing protease inhibitors [34,36].

Old age, increased body mass index, hypercholesterolemia, hypertriglyceridemia contributes to increased risk of insulin resistance. Patients with these risk factors or with pre-existing diabetes mellitus should be monitored. Knowledge of early symptoms such as polydipsia, polyphagia and/or polyuria are beneficial in the rapid initiation of appropriate treatment. However, data from published literature are not enough to say whether the treatment with protease inhibitors should be discontinued in patients with newly diagnosed diabetes mellitus [37].

Comparing the values of the biochemical parameters specific to lipid metabolism, the study showed that the mean incidence of the increased level of total cholesterol (NV 140–220 mg/dL) is 1.53 with SD 0.49. It was observed that all analyzed parameters show a slightly increased frequency of values above normal, i.e., of decreasing values (for HD-cholesterol) for patients in group 1 (DRV/r) relative to group 2 (DRV/c). The same result was obtained from a multi-center study carried out between December 2015 and May 2016 [38].

Although there are differences between the two groups, they did not reach the threshold of statistical significance (*p* < 0.05), which indicates that all patients in the cohort have some degree of impairment of lipid metabolism, regardless the regimen. Previous studies have shown that hyper triglycerides and hyper LDL-cholesterol, associated with low levels of HDL-cholesterol, increase the risk of cardiovascular diseases [39,40,41]. Studies before do not established exactly how the disorders in lipid metabolism are influenced by ART, but it seems that the PIs binds to proteins involved in the regulation of lipid metabolism, low-density lipoprotein-receptor protein (LRP) and cytoplasmic retinoic acid binding protein type 1 (CRABP1), which are 60% similar to HIV proteins [42]. On the other hand, it has been demonstrated that the treatment with NRTI affects mitochondrial function by inhibiting the transcription and mitochondrial DNA depletion [43].

The association of these two classes (PI and NRTI) in HIV^+^ patients’ regimens imply a high level of risk. A study carried out on mice model has shown that PI, even at low doses, which do not cause dyslipidemia, still induce the emergence and progression of atherosclerotic lesions. Increasing PI doses could lead to greater extension of atherosclerotic lesions, even if no direct link has been demonstrated [44].

A study carried out on healthy volunteers who have taken RTV, showed an increase in TC, TG, and VLDL-cholesterol levels, 14 days after administration [45]. Another study also showed that NRTI, efavirenz, associated with a PI, amprenavir, in the regimen caused an increases serum lipid levels, which persisted up to five weeks [46].

A prospective observational study from 2007 highlighted a higher incidence of myocardial infarction in patients treated with protease inhibitors, but which decreased after the normalization of lipidemia. The same results were found in experienced patients, therapeutically aged [47]. Therefore, the choice of an antiretroviral regimen with a few side effects on lipid metabolism remains a challenge. Even small, apparently insignificant differences are to be considered and can tilt the balance at least in terms of adherence to treatment.

Regarding lipodystrophy syndrome, the results obtained, analyzing the two groups, were approximately equal, with 35.4% of the DRV/r group and 33.3% of the DRV/c group having this diagnosis. The mean value of lipodystrophy in DRV/r is slightly higher than at DRV/c, which shows that lipodystrophy prevalence as the pre-existing disease (before the current regimen) is higher at DRV/r. The small difference between the two groups indicates that the presence of the lipodystrophy syndrome cannot be linked to exposure to one or other of the enzyme inhibitors and should be correlated with other parameters. Studies have reported the presence of this metabolic disorder with different prevalence between 2% and 84% among PLWH [48]. The difference arises from the unclear definition of the syndrome. There are several diseases included in this condition: lipoatrophy, lipo-hypertrophy, mixed syndrome [49]. The association of different types of ARV drugs leads to various lipodystrophy changes.

So far, studies have shown that the occurrence of this syndrome is, primarily, related to the prolonged use of antiretroviral drugs, two classes being more frequently involved in its onset: PI and NRTI [50,51,52]. The FRAM study pointed out that NNRTI are responsible for the reduction of subcutaneous fat tissue in the legs, particularly in women, while in men this link is not so well expressed [53]. Lipodystrophy, whether caused by infection itself or by ART, is an important additional factor in the emergence of cardiovascular risk to which HIV patients are exposed.

As for dyslipidemia, the prevalence is higher among subjects in the first group, DRV/r (28.6%) compared to the second, DRV/c (15.3%). Dyslipidemia is frequently observed in HIV-infected patients and is linked to increases in triglycerides, decreased HDL-cholesterol level and variable increases in LDL-cholesterol and TC. However, the cause-effect relationship between dyslipidemia and specific antiretroviral agents has not been clearly emphasized [54]. Its pathogenesis is complex and is related with the virus, the host and with antiretroviral treatment. Dyslipidemia is a major cardiovascular risk factor and can be partially treated. Because HIV infection and its treatment are linked with progression of atherosclerosis and an increased number of cases of myocardial infarction, the management of dyslipidemia must be a priority in clinical care of patients with HIV infection. Previous studies have made recommendations on the management of this disease. These involve the control of other cardiovascular risk factors, choosing an antiretroviral drug with a better lipid profile and the use of lipid lowering drugs, when clinically indicated [55]. Dietary interventions (low fat diet), change of lifestyle, including exercise in the daily routine should be added to all these pharmacological interventions [56]. Regarding preexisting conditions, the prevalence of diabetes, lipodystrophy, and dyslipidemia was statistically correlated. The results of this study, with statistical significance, have shown that experienced aged patients are more likely to develop disorders of lipid metabolism, such as lipodystrophy and dyslipidemia. The same has been demonstrated by previous studies [57]. The greater the number of comorbidities, the number of treatments past regimens and pill’s burden, the higher the risk of dyslipidemia. The risk of suffering from lipodystrophy is higher in patients with a greater number of associated diseases. Previous studies have already established that ART, especially in the first generations, although improves the patient’s clinical condition, can lead to several metabolic disorders, the most common being diabetes, lipodystrophy, and dyslipidemia [58]. Despite the new antiretroviral drugs, patients previously treated with old regimens, which mainly contained NRTI, continue to experience long-term metabolic alterations [59].

The proper function of the cardiovascular system is directly related to carbohydrate and lipid metabolism. This study demonstrated that the mean values of CK, CK-MB, fibrinogen, and PT/INR show an increase in the DRV/r group, implying a more affected cardiovascular function with a higher risk of developing cardiovascular disorders compared to patients in the DRV/c group. This is also confirmed by the fact that although in both cases the range values are normal, there is a significant difference between the two groups. Statistically correlating the values of the cardiac and coagulation parameters with demographic and disease-specific characteristics, the study observed a statistically significant correlation (*p* < 0.05) between the fibrinogen value and the age. Aging is an additional risk factor in the increase of coagulation factor I serum level, a marker of a deteriorated cardiac tissue. It is proven that the mortality rate through coronary disease, the acute risk of myocardial infarction and the risk of ischemic stroke increase with age, among PLWH [60,61].

The metabolic disorders in lipid profile, evaluated in the present study entail an increased risk of atherosclerosis, which is known as endothelial dysfunction. High levels of LDL-cholesterol, the free radicals produced by cigarette smoke, diabetes, and hypertension were included among the drivers of endothelial dysfunction [62]. In addition, HIV has been shown to interfere with the endothelial membrane cells by initiating inflammatory and intracellular biochemical reactions leading to the subsequent emergence of atherosclerosis [63]. The cause of these disorders can also be correlated with the duration of HIV infection but can also be an effect of ART [64]. A favorable factor is the therapy with protease inhibitors. In a large study on 20,000 patients, published in 2003, it has been shown that the use of PI alone or in association with NNRTI, especially in multi-experienced patients, increases the risk of cardiac function impairment, through changes in lipid metabolism. The study observed an additive effect of side effects produced by drugs from different classes. The greater the burden of the pill, the greater the number of risk factors [65].

RTV damages mitochondrial DNA in endothelial cells, leading to cell death. The decrease in endothelial cell viability and increase toxicity depend on the dose and on the duration of exposure to the RTV treatment [66]. Most studies associate the increased risk of cardiovascular disease with ART’s duration. They emphasized the importance of early treatment and identification of patients’ risk profiles [67,68].

Exposure to DRV boosted with RTV is more frequently associates with the risk of hypercholesterolemia, hypertriglyceridemia, and hyperlipidemias. As giving up antiretroviral medication is not an option, less aggressive regimens for lipid metabolism should be selected for patients with high cardiovascular risk.

The results of this study showed no changes in analyzed coagulation markers (PT/INR and fibrinogen); however, a higher fluctuation of the values is found in the first group and higher values. Previous studies have shown that, even though the infection can be controlled under ART, the activation of hemostasis and fibrinolysis may occur with the duration of ART [69,70]. Chronic HIV infection makes changes in hemostasis and coagulation as consequence of persistent systemic immune activation, micro and macro-vascular diseases and due to affected liver syntheses of coagulation factors [71]. The aging process occurs earlier in HIV infected people, because of multiple causes regarding the infection itself, but also independently of it [72]. Studies have confirmed that pre-thrombotic status is associated with comorbidities and the disease stage [73]. When these two are associated, the risk of mortality increases and the survival rate decreases, as the PLWH decreases [74].

Looking at the patient’s risk profile of suffering various disorders in the main human body systems or organs, this study showed that the risk of cardiovascular disease increases, with the patient’s aging, the age of infection, the number of co-morbidities and the number of associated ARV’s. If the first parameters cannot be changed, the burden of pill can be managed by choosing efficient but simplified regimens. The results have shown the following statistical significance: the level of TC is strongly positively correlated with age—the older the patient, the higher the serum level of TC; whereas HDL-cholesterol correlates negatively with the number of years of therapy—the longer the infection lasts, the lower the serum level of HDL-cholesterol. The same type of relationship, inversely proportional is observed in relation to the number of ARVs in regimen. Low levels of HDL-cholesterol can be observed at treatment multi-experienced patients. Positive correlation, a directly proportional increase, exists between the level of LDL-cholesterol and the number of comorbidities—the more associated diseases there are, the greater levels of low-density cholesterol serum levels. TL correlate directly proportionally, strongly positive (*p* < 0.01) to age–the level of TL increases with aging and at a threshold of *p* < 0.05 with the number of years of therapy. The VLDL-cholesterol level increases with aging, number of years of therapy and number of regimens (strongly positive correlation *p* < 0.01), but also to the number of comorbidity (positive correlation *p* < 0.05). Strongly positive correlation also exists between glycosylated hemoglobin and age, number of years of therapy, comorbidity, number of ARVs. The increase of the latter leads to a directly proportional increase in the level of glycosylated hemoglobin, thus a greater risk of developing diabetes. The value of blood sugar has been significantly correlated, strongly positive *p* < 0.01, with the patient’s age alone, with an increased risk of diabetes mellitus.

For liver function, the mean values for ALT, AST, GGT, and ALP but also total bilirubin and direct bilirubin show higher values for DRV/r patients, which indicates increased tolerance for patients in the DRV/c group. The study has shown that more patients among those exposed to RTV, as pharmacokinetic enhancer, had increases parameters values, directly related to liver damage. The enzyme inhibitor plays a very important role, as it influences the metabolism of drugs. Previous studies had established that liver function tests, for PLWH, can be negatively affected by both antiretroviral therapy and coinfections with hepatitis B or C virus or by alcohol abuse. Studies have shown that the tests’ results change, regardless of the regimen used, in 2% to 18% of patients [75,76]. A possible cause of liver damage is the immune reconstitution inflammatory syndrome following the initiation of ART. Studies emphasize that patients under antiretroviral therapy should be monitored regularly for their liver parameters [77]. The moment when liver damage occurs can be predicted depending on which antiretroviral classes is used. The study carried out by Carr et al. [74] states that nucleoside analogues lead to hepatic steatosis probably caused by mitochondrial toxicity occurring within six months of treatment. NNRTI causes hypersensitivity reaction within three months of administration. Liver function tests should be performed monthly for patients with pre-existing liver disorders and every three months for others. If the value of liver enzyme is in the moderately high range, <3.5 times the normal upper limit, ALT–30 IU/L and AST–25 IU/L, in the absence of clinical symptoms, treatment can be continued under monitoring. Higher increases in liver enzymes require further analysis, including imaging and determination of drug plasma concentrations. For patients with VHB and/or VHC, the specific treatment for the viral hepatitis infections should also be taken into account as the effectiveness of ART may be reduced and hepatic toxicity may increase [78].

The results of the comparative study, regarding the degree of the pancreatic function impairment, showed that most patients in the two groups have normal values of the serum levels of amylase and lipase. Although in both groups there are patients above the reference range, the balance tilts against those exposed to the DRV/r, thus the risk of liver damage is higher for these subjects. Previous studies have shown that hyperamylasemia and hyperlipasemia are caused by pancreatic disorders, usually pancreatitis, or extra-pancreatic diseases—gastro-intestinal, renal, acidosis, parotid disorders [79,80].

The growth of pancreatic enzymes may not be linked with pancreatitis. Mild and moderate increases are common in HIV^+^ patients and have been associated with positive serology for chronic B or C hepatitis and with other drugs, especially antiretroviral or intravenous cotrimoxazole [81]. Many studies have reported increased lipase and amylase serum values [82,83]. A direct causal link between serum increases in liver enzymes and antiretroviral therapy could not be demonstrated in any of the studies cited above.

For liver function, statistically significant correlations have shown that the VHC co-infection influences the increase in liver enzyme levels, to a greater extend. The number of comorbidity and ARV’s also expose the patient to a higher risk of liver damage. ALT decreases with the patient’s number of past regimens. Strong positive correlation (*p* < 0.01) exists with the number of comorbidities and the presence of VHC co-infection and positive, *p* < 0.05, with the burden of pills. Therefore, the ALT level increases in VHC co-infected patients with multiple comorbidities and with more pills administered. Levels of hepatic AST and GGT have a statistically significant correlation with the presence of VHC infection (*p* < 0.01), which represents an additional risk factor for the growth of serum levels of these enzymes. The total and direct bilirubin levels meet the statistical significance when correlated with VHC co-infection. The presence of VHC leads to higher values of the two parameters. They also increase in patients co-infected with VHB or with aging, the duration of ART, number of regimens, comorbidities, or number of associated ARVs but without statistical significance. For pancreatic enzymes, statistically significant results have been obtained only when correlating the serum levels of lipase. It increases proportionally, statistically significantly (*p* < 0.05) with age and duration of ART and have a highly positive correlation (*p* < 0.01) with the number of past regimens, to which the patients had been exposed.

For all patients in the cohort, the mean value of renal function markers–urea, creatinine, uric acid, and creatinine clearance is within normal reference ranges. However, comparing the two groups, the results of the study showed a better profile for DRV/c group. Most patients with outcomes above reference ranges and with the highest fluctuations of values were found in patients taking DRV/r. Therefore, although average values for serum urea are relatively close, a higher risk of an impaired kidney function can be observed for patients exposed to the DRV/r. For serum creatinine, comparative statistics, between the two groups, show a significant difference. This indicates a better tolerability to boosting PI with COBI. Applying MDRM equation we obtained similar results for the subjects in the two groups, for the first CKD stages (1, 2, and 3A). Closer monitoring of renal markers is required because patients may accuse signs of proteinuria or other lesions. For patients in 3A CKD stage, kidney damage is moderate, hand and foot are sweating, back pain, and urination above or below normal occur. Advanced CKD stages, with moderate and severe symptoms (3B, 4, and 5), have only been observed in patients from the first group, DRV/r. Other symptoms may appear at these stages: anemia, increase in blood pressure, bone disease, cramps, muscle pain, lack of appetite, edema and, in the end, dialysis. Similar results were obtained in another study carried out on a cohort in Romania, that was administrated another PI, lopinavir, boosted also with RTV [84].

By correlating the creatinine clearance with the incidence of CKD stages, the study showed a strong negative relation—the lower the glomerular filtration capacity, the higher the risk of developing severe kidney damage. The higher number of patients from the first group (DRV/r) with all CKD stages, indicates a lower tolerability for this regimen. Previous studies have been demonstrated that conditions associated with increased serum urea, called azotemia, may occur in acute or CKD, glomerulonephritis, skin disease, urinary tract obstruction, drop in renal perfusion. Hyperuricemia may be a sign of chronic renal failure or may be associated with other diseases: diabetes, leukemia, neoplasms, or infectious diseases. Despite the obvious benefits of ART in suppressing viral replication, some ARVs may occasionally induce reversible or irreversible renal lesions [31]. Studies talk about kidney damage for almost all antiretroviral agents that are, now, used in therapy. However, situations of renal toxicity remain exceptions [85].

Different types of nephrotoxicity have been reported in patients treated with reverse transcriptase nucleoside inhibitors (NRTI), non-nucleosidase inhibitors (NRTI), or protease inhibitors (PI). Pathogenetic mechanism of kidney lesions remains unknown. Three antiretroviral agents may be directly associated with renal toxicity: tenofovir, indinavir, and atazanavir [86,87,88,89].

A meta-analysis of the relative risks of kidney disease among HIV^+^ patients has shown that they are at significantly higher risk of developing a kidney disease than people who are not infected with HIV. Even so, HIV^+^ patients receiving ART are less likely to develop renal diseases than naive patients, who do not take therapy [90].

In various studies, a correlation has been made between certain ARVs and a certain segment of the kidney which can be damaged: tenofovir, didanosine for glomerular dysfunction [88]; tenofovir, didanosine, abacavir, PI boosted with RTV—proximal tube dysfunction [89]; stavudine, didanosine, lamivudine–tubular acute necrosis [90]; abacavir, efavirenz, atazanavir, indinavir—acute interstitial nephritis [90]; indinavir, atazanavir, rarely other PI—obstructive uropathy [90]; zidovudine, didanosine—pigmented glomerular nephropathy [84]; stavudine, didanosine, PI boosted with RTV—chronic vascular disease [90].

CKD has become a common comorbidity among experienced HIV-infected patients, even though ART is responsible for prolonging their life expectancy. The causes of nephrotoxicity must be investigated to determine to what extent this is caused by ARVs, the disease itself, or by associated drugs. The present study showed that: serum levels of urea, creatinine, and uric acid tend to be higher—above laboratory references ranges—with age, the increase being directly proportional. The level of uric acid also increases in direct proportion to the number of comorbidities, (*p* < 0.01). The greater the number of comorbidities, the greater the level of serum, uric acid. The duration of ART appears to be responsible for low creatinine clearance values (strongly negative correlation) and advanced CKD stages (strongly positive correlation). The more experienced the patient, the more severe the glomerular impairment. Thus, the risk of serious kidney damage increases.

Extensive, randomized studies are needed to evaluate the long-term renal consequences of ARVs and to determine which serum markers are the most sensitive in screening for renal abnormalities. Periodic monitoring of the renal function is one of the clinicians’ tools to minimize renal side effects.

## 5. Conclusions

The results of this study have shown that DRV/c containing regimens, appear to be better tolerated and with a lower impact on metabolic profiles than DRV/r containing regimens. The use of COBI as pharmacokinetic enhancer in HIV^+^ patients’ regimens, experienced or naïve, has advantages in long-term therapy. The results of this study can be useful in guiding the clinicians in the management of antiretroviral therapy, highlighting the need for a personalized therapy (tailoring medicine) that considers a patient’s response to therapy, their therapeutic history, associated medicines, disease history, and comorbidities.

## Figures and Tables

**Table 1:** Demographic and clinical characteristics.

Variable		DRV/r		DRV/c		Total	
		n	%	n	%	N	%
**Demographic data**							
Number n (%)		384	83.11	78	16.89	462	100
Female n %		148	38.50	28	35.90	176	38.09
Male n %		236	61.50	50	61.50	286	61.91
Mean age. years (±SD)		39.41	±11.50	38.58	±10.45	39.27	11.32
t		74.544					
*p*		0.001					
**Characteristics of the disease**							
HIV-1 RNA (copies/mL)							
Mean (±SD)		1.56	±1.04	1.38	±0.93	1.53	±1.02
t		32.081					
*p*		0.007					
Undetectable (=0) n %		292	76.6	68	84.61	358	78.0
<50		0	0.0	0	0.0	0	0.0
50–999		55	14.4	6	7.6	61	13.3
>1000		34	8.9	6	7.6	40	8.7
CD4 T-cells (cells/mm^3^)							
Mean (±SD)		3.21	±1.01	3.35	±0.91	3.23	±1.00
t		69.830					
*p*		0.001					
0–199		37	9.6	5	6.4	42	9.1
200–349		53	13.8	8	10.3	61	13.2
350–499		86	22.4	20	25.6	106	22.9
>500		208	54.2	45	57.7	253	54.8
CDC’s HIV-1 classes							
Mean (±SD)		6.21	±3.5	5.17	±3.81	6.04	±3.5
t		36.380					
*p*		0.001					
Unknown		63	16.4	19	24.3	82	17.7
A1		8	2.1	3	3.8	11	2.4
A2		15	3.9	5	6.3	20	4.3
A3		11	2.9	3	4.0	14	3.0
B1		2	0.5	0	0.0	2	0.4
B2		33	8.6	8	10.2	41	8.9
B3		34	8.9	5	6.4	39	8.4
C1		3	0.8	0	0.0	3	0.6
C2		18	4.7	4	5.1	22	4.8
C3		197	51.3	31	39.8	228	49.4
Mean duration of the infection years (±SD)		11.05	±9.16	9.17	±8.19	10.74	(±9.02)
t		25.576					
*p*		0.001					
Duration of the infection years (%)	Unknown	60	15.6	6	7.7	66	14.3
	0–5	78	20.3	31.9	39.7	109	23.6
	6–10	59	15.4	12	15.3	71	15.4
	11–20	111	28.9	18	23.2	129	27.9
	21–30	75	19.5	11	14.1	86	18.6
	>30	1	0.3	0	0.0	1	0.2
Mean duration of the ART years (±SD)		11.17	±6.15	9.57	±6.37	10.90	±6.21
t		37.699					
*p*		0.001					
Duration of the ART years (%)	naive	95	24.7	30	38.5	125	27.1
	1–5	98	25.5	21	26.9	119	25.7
	6–10	191	49.8	27	34.6	218	47.2
	11–15	0	0.0	0	0.0	0	0.0
	16–20	0	0.0	0	0.0	0	0.0
Mean number of ARV. pills (±SD)		3.48	±0.57	2.38	±0.54	3.29	±0.70
t		101.166					
*p*		0.001					
Mean number of therapeutic regimens (±SD)		9.09	±7.42	7.89	±6.11	8.88	±7.23
t		26.423					
*p*		0.001					
Naive		28	7.3	2	2.56	30	6.5
Experienced		356	92.7	76	97.43	432	93.5

Legend: SD—standard deviation; t—Student’s *t*-test coefficient t; *p*—statistical significance.

**Table 2:** Drugs included in the regimens.

DRV/r (n = 384)			DRV/c (n = 78)		
Regimen	Patients		Regimen	Patients	
	n	%		n	%
ABC + 3TC + DRV + RTV	151	39.33	ABC + 3TC + DRV + COBI	37	47.43
FTC + TDF + DRV + RTV	46	11.97	FTC + TDF + DRV + COBI	18	23.07
3TC + ZDV + DRV + RTV	34	8.85	3TC + ZDV + DRV + COBI	7	8.97
ETV + RAL + DRV + RTV	17	4.42	TDF + DTG + DRV +COBI	4	5.12
TDF + ETV + DRV + RTV	17	4.42	FTC + TAF + DRV + COBI	3	3.84
TDF + DTG+ DRV + RTV	14	3.64	ETV + RAL + DRV +COBI	2	2.56
RAL + DRV+ RTV	11	2.86	RAL + DRV + COBI	2	2.56
Others	94	24.47	Others	5	6.41

Legend: ABC—abacavir; COBI—cobicistat; DTG—dolutegravir; DRV—darunavir; ETV—etravirine; FTC—emtricitabine; RAL—raltegravir; RTV—ritonavir; TAF—tenofovir alafenamide; TDF—tenofovir disoproxil fumarate; ZDV—zidovudine; 3TC-lamivudine.

**Table 3:** Descriptive statistics of lipid profile parameters (*p* < 0.01).

Variables		DRV/r n = 384	%	DRV/c n = 78	%	Total N = 462	%	*t*
TC	1	176	45.8	38	48.7	214	46.3	66.172
	2	208	54.2	40	51.3	248	53.7	
HDL-cholesterol	0	134	34.9	10	12.8	144	31.2	31.907
	1	250	65.1	68	87.2	318	68.8	
LDL-cholesterol	1	160	41.67	39	50	199	43.08	68.043
	2	224	58.33	39	50	263	56.92	
TG	1	168	43.8	44	56.4	212	45.9	66.404
	2	216	56.2	34	43.6	250	54.1	
TL	1	247	64.3	61	78.2	308	66.7	60.729
	2	137	35.7	17	21.8	154	33.3	
VLDL-cholesterol	1	301	78.4	71	91	372	80.5	2.583
	2	83	21.6	7	9	90	19.5	

Legend: 1—normal values, 2—increased values, N—number of patients in cohort, n—number of patients in group.

**Table 4:** Pearson correlation on the risk of impaired carbohydrate and lipid metabolism in HIV^+^ patients (N = 462).

Variables	CT	HDL	LDL	TG	LT	VLDL	Glycosylated Hemoglobin	Glycemia
Age	r = 0.207 **	r = 0.008	r = 0.069	r = 0.240 **	r = 0.221 **	r = 0.206 **	r = 0.324 **	r = 0.216 **
	(*p* = 0.001)	(*p* = 0.878)	(*p* = 0.139)	(*p* = 0.001)	(*p* = 0.001)	(*p* = 0.001)	(*p* = 0.001)	(*p* = 0.001)
Duration of ART	r = 0.052	r = −0.140 **	r = 0.006	r = 0.040	r = 0.093 *	r = 0.182 **	r = 0.189 **	r = 0.002
	(*p* = 0.263)	(*p* = 0.003)	(*p* = 0.906)	(*p* = 0.393)	(*p* = 0.045)	(*p* = 0.001)	(*p* = 0.001)	(*p* = 0.974)
Number of ARV regimens	r = 0.072	r = −0.026	r = 0.019	r = 0.004	r = 0.075	r = 0.130 **	r = 0.046	r = 0.029
	(*p* = 0.121)	(*p* = 0.583)	(*p* = 0.682)	(*p* = 0.930)	(*p* = 0.110)	(*p* = 0.005)	(*p* = 0.325)	(*p* = 0.534)
Number of comorbidities	r = −0.064	r = −0.079	r = 0.113 *	r = 0.028	r = 0.051	r = 0.104 *	r = 0.123 **	r = −0.012
	(*p* = 0.170)	(*p* = 0.089)	(*p* = 0.015)	(*p* = 0.545)	(*p* = 0.278)	(*p* = 0.026)	(*p* = 0.008)	(*p* = 0.793)
Pill’s burden	r = −0.059	r = −0.120 **	r = 0.020	r = 0.068	r = 0.066	r = 0.107 *	r = 0.133 **	r = 0.015
	(*p* = 0.207)	(*p* = 0.010)	(*p* = 0.673)	(*p* = 0.144	(*p* = 0.158)	(*p* = 0.021)	(*p* = 0.004)	(*p* = 0.756)

Legend: N—number of patients in cohort; r—Pearson’s ratio; *p*—statistical significance; *—statistical significance *p* < 0.05; **—statistical significance *p* < 0.01; negative values indicate the inversely proportional correlation between the two parameters concerned; positive values show a directly proportional correlation.

**Table 5:** Descriptive statistics: prevalence of lipodystrophy and dyslipidemia (*p* < 0.01).

Variables	DRV/r n = 384		DRV/c n = 78		Total N = 462		
	Mean	SD	Mean	SD	Mean	SD	*t*
Lipodystrophy	0.3542	0.47888	0.3333	0.47446	0.0173	0.13059	2.850
Dyslipidemia	0.2630	0.44085	0.1538	0.36314	0.3506	0.47769	15.778

Legend: N—number of patients in cohort; n—number of patients in group; SD—standard deviation.

**Table 6:** Pearson correlation on the risk of developing lipodystrophy or dyslipidemia for all the patients (N = 462).

Variables	Diabetes	Lipodystrophy	Dyslipidemia
Age	r = 0.009	r = 0.185 **	r = 0.165 **
	(*p* = 0.851)	(*p* < 0.001)	(*p* < 0.001)
Duration of ART	r = 0.008	r = 0.127 **	r = 0.272 **
	(*p* = 0.872)	(*p* = 0.006)	(*p* = 0.000)
Number of ARV regimens	r = 0.010	r = 0.035	r = 0.109 *
	(*p* = 0.838)	(*p* = 0.453)	(*p* = 0.019)
Number of comorbidities	r = −0.048	r = 0.105 *	r = 0.346 **
	(*p* = 0.299)	(*p* = 0.025)	(*p* < 0.001)
Pill’s burden	r = −0.041	r = 0.039	r = 0.101 *
	(*p* = 0.378)	(*p* = 0.397)	(*p* = 0.030)

Legend: N—number of patients in cohort; r—Pearson’s ratio; *p*—statistical significance; *—statistical significance *p* < 0.05; **—statistical significance *p* < 0.01; negative values indicate the inversely proportional correlation between the two parameters concerned; positive values show a directly proportional correlation.

**Table 7:** Descriptive statistics of cardiac and coagulation markers (*p* < 0.01).

Variables	DRV/r n = 384		DRV/c n = 78		Total N = 462		
	Mean	SD	Mean	SD	Mean	SD	*t*
CK	110.57	237.94	80.29	67.72	105.46	218.93	10.353
CK-MB	14.43	15.64	14.23	12.47	14.40	15.14	20.443
PT/INR	1.03	0.22	1.02	0.10	1.03	0.20	109.963
Fibrinogen	325.45	104.58	319.27	102.59	324.41	104.17	66.940

Legend: N—number of patients in cohort; n—number of patients in group; SD—standard deviation.

**Table 8:** Pearson correlation on the risk of heart markers and coagulation in HIV^+^ cohort patients (N = 462).

Variables	CK	CK-MB	PT/INR	Fibrinogen
Age	r = −0.036	r = −0.051	r = 0.058	r = 0.180 *
	(*p* = 0.440)	(*p* = 0.276)	(*p* = 0.215)	(*p* = 0.001)
Duration of ART	r = −0.030	r = 0.024	r = −0.052	r = −0.029
	(*p* = 0.525)	(*p* = 0.614)	(*p* = 0.268)	(*p* = 0.538)
Number of ARV regimens	r = −0.011	r = 0.084	r = −0.008	r = 0.074
	(*p* = 0.525)	(*p* = 0.071)	(*p* = 0.860)	(*p* = 0.111)
Number of comorbidities	r = −0.012	r = −0.011	r = 0.040	r = 0.077
	(*p* = 0.799)	(*p* = 0.810)	(*p* = 0.389)	(*p* = 0.100)
Pill’s burden	r = −0.018	r = 0.046	r = −0.018	r = 0.087
	(*p* = 0.701)	(*p* = 0.319)	(*p* = 0.692)	(*p* = 0.063)

Legend: N—number of patients in cohort; r—Pearson’s ratio; *p*—statistical significance; * —statistical significance *p* < 0.01; negative values indicate the inversely proportional correlation between the two parameters concerned; positive values show a directly proportional correlation.

**Table 9:** Descriptive statistics of liver function parameters (*p* < 0.01).

Variables	DRV/r n = 384		DRV/c n = 78		Total N = 462		
	Mean	SD	Mean	SD	Mean	SD	*t*
ALT/GPT	36.62	38.34	29.02	15.56	35.34	35.64	21.313
AST/GOT	34.94	31.17	29.07	10.38	33.95	28.81	25.331
GGT	54.55	140.34	36.48	29.22	51.50	128.65	8.605
ALP	82.69	34.93	76.96	26.31	81.72	33.67	52.166
Total bilirubin	0.75	1.34	0.70	0.64	0.74	1.25	12.812
Direct bilirubin	0.28	0.41	0.26	0.15	0.28	0.38	16.071
Lipase	126.73	100.67	124.44	137.04	126.35	107.50	25.262
Amylase	73.91	35.01	65.85	18.54	72.55	32.94	47.338

Legend: N—number of patients in cohort; n—number of patients in group; SD—standard deviation.

**Table 10:** Pearson correlation on the risk of liver function in HIV positive patients (N = 462).

Variables	ALT/GPT	AST/GOT	GGT	Total Bilirubin	Direct Bilirubin	Lipase	Amylase
Age	r = −0.071	r = −0.042	r = 0.034	r = 0.018	r = 0.005	r = 0.092 *	r = −0021.
	(*p* = 0.129)	(*p* = 0.371)	(*p* = 0.465)	(*p* = 0.700)	(*p* = 0.922)	(*p* = 0.047)	(*p* = 0.651)
Duration of ART	r = −0.052	r = −0.059	r = −0.032	r = 0.018	r = 0.010	r = 0.120 *	r = 0.089
	(*p* = 0.263)	(*p* = 0.204)	(*p* = 0.492)	(*p* = 0.359)	(*p* = 0.833)	(*p* = 0.010)	(*p* = 0.057)
Number of ARV regimens	r = −0.105 *	r = −0.021	r = 0.006	r = 0.043	r = −0.026	r = −0.114 **	r = −0.006
	(*p* = 0.024)	(*p* = 0.660)	(*p* = 0.890)	(*p* = 0.696)	(*p* = 0.575)	(*p* = 0.014)	(*p* = 0.901)
Number of comorbidities	r = 0.165 **	r = 0.072	r = 0.032	r = 0.015	r = 0.046	r = −0.047	r = −0.012
	(*p* = 0.001)	(*p* = 0.124)	(*p* = 0.494)	(*p* = 0.752)	(*p* = 0.322)	(*p* = 0.318)	(*p* = 0.795)
Pill’s burden	r = 0.118 *	r = 0.069	r = 0.016	r = 0.030	r = 0.050	r = −0.040	r = −0.024
	(*p* = 0.011)	(*p* = 0.141)	(*p* = 0.733)	(*p* = 0.522)	(*p* = 0.283)	(*p* = 0.389)	(*p* = 0.609)
VHC co-infection	0.200 **	r = 0.194 **	r = 0.194 **	r = 0.102 *	r = 0.201 **	r = −0.050	r = −0.011
	(*p* = 0.001)	(*p* = 0.001)	(*p* = 0.001)	(*p* = 0.028)	(*p* = 0.001)	(*p* = 0.285)	(*p* = 0.817)
VHB co-infection	r = 0.063	r = 0.018	r = −0.012	r = 0.040	r = 0.056	r = −0.016	r = −0.019
	(*p* = 0.178)	(*p* = 0.694)	(*p* = 0.794)	(*p* = 0.393)	(*p* = 0.227)	(*p* = 0.738)	(*p* = 0.686)

Legend: N—number of patients in cohort; r—Pearson’s ratio; *p*—statistical significance; *—statistical significance *p* < 0.05; **—statistical significance *p* < 0.01; negative values indicate the inversely proportional correlation between the two parameters concerned; positive values show a directly proportional correlation.

**Table 11:** Descriptive statistics of renal parameters.

Variables	DRV/r n = 384		DRV/c n = 78		Total N = 462		
	Mean	SD	Mean	SD	Mean	SD	*t*
Urea	33.2544	18.61	32.5218	8.60	33.1307	17.32	41.093 *
Creatinine	0.8797	0.67	0.8564	0.20	0.8758	0.61	30.404 *
Uric acid	5.0146	1.67	5.3192	1.26	5.0660	1.61	67.534 *
Creatinine clearance	98.90	30.45	96.46	23.06	98.49	29.33	72.176 *

Legend: N—number of patients in cohort; n—number of patients in group; SD—standard deviation; *—statistical significance *p* < 0.01.

**Table 12:** Pearson correlation on the risk of impaired renal function in HIV positive patients (N = 462).

Variables	Urea	Creatinine	Uric Acid	Creatinine Clearance	Stages of CKD
Age	r = 0.189 **	r = 0.091 *	r = 0.148 **	r = 0.056	r = 0.024
	(*p* = 0.001)	(*p* = 0.050)	(*p* = 0.001)	(*p* = 0.228)	(*p* = 0.611)
Duration of ART	r = −0.008	r = −0.044	r = −0.015	r = −0.239 **	r = 0.255 **
	(*p* = 0.867)	(*p* = 0.340)	(*p* = 0.748)	(*p* = 0.000)	(*p* = 0.000)
Number of ARV regimens	r = 0.032	r = −0.012	r = −0.065	r = 0.003	r = 0.006
	(*p* = 0.491)	(*p* = 0.801)	(*p* = 0.163)	(*p* = 0.953)	(*p* = 0.901)
Number of comorbidities	r = 0.036	r = −0.011	r = 0.138 **	r = 0.040	r = −0.042
	(*p* = 0.445)	(*p* = 0.818)	(*p* = 0.003)	(*p* = 0.389)	(*p* = 0.372)
Pill’s burden	r = 0.028	r = 0.043	r = −0.046	r = 0.023	r = 0.010
	(*p* = 0.453)	(*p* = 0.352)	(*p* = 0.326)	(*p* = 0.618)	(*p* = 0.827)

Legend: N—number of patients in cohort; r—Pearson’s ratio; *p*—statistical significance; *—statistical significance *p* < 0.05; **—statistical significance *p* < 0.01; negative values indicate the inversely proportional correlation between the two parameters concerned; positive values show a directly proportional correlation.

## Data Availability

Data can be found in the archive data base of “Prof. Dr. Matei Bals” National Institute of Infectious Diseases, Bucharest, Romania.
